# Acidic Microenvironment Up-Regulates Exosomal miR-21 and miR-10b in Early-Stage Hepatocellular Carcinoma to Promote Cancer Cell Proliferation and Metastasis: Erratum

**DOI:** 10.7150/thno.60140

**Published:** 2021-04-20

**Authors:** Xiao-Peng Tian, Chen-Yuan Wang, Xiao-Han Jin, Mei Li, Feng-Wei Wang, Wei-Juan Huang, Jing-Ping Yun, Rui-Hua Xu, Qing-Qing Cai, Dan Xie

**Affiliations:** 1 Sun Yat-sen University Cancer Center, State Key Laboratory of Oncology in South China, Collaborative Innovation Center of Cancer Medicine, Guangzhou, China; 2 Department of Reproductive Medicine, The Second Affiliated Hospital of Guangzhou University of Chinese Medicine, Guangzhou, China.; 3 Department of Pathology, Sun Yat-sen University Cancer Center, Guangzhou, China; 4 Department of Pharmacology, College of Pharmacy, Jinan University, Guangzhou, China; 5 Department of Medical Oncology, Sun Yat-sen University Cancer Center, Guangzhou, China

In our article, there were one misplaced image in Figure 5E and Figure 5I, respectively. The corrected version is provided here:

The correction made in this erratum does not affect the original conclusions. The authors apologize for any inconvenience or misunderstanding that this error may have caused.

## Figures and Tables

**Figure 5:**
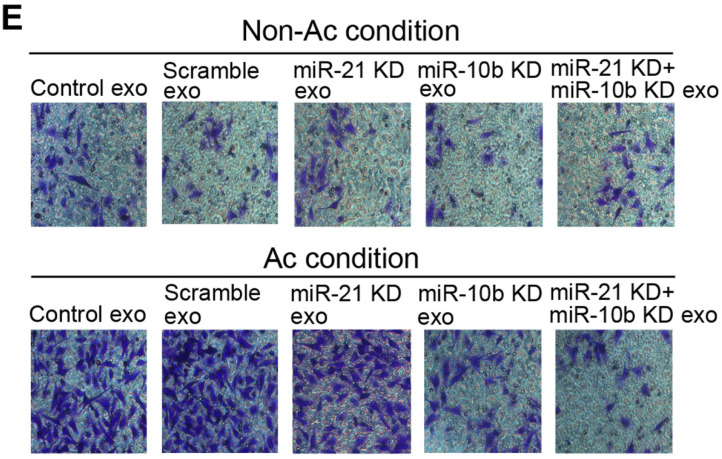

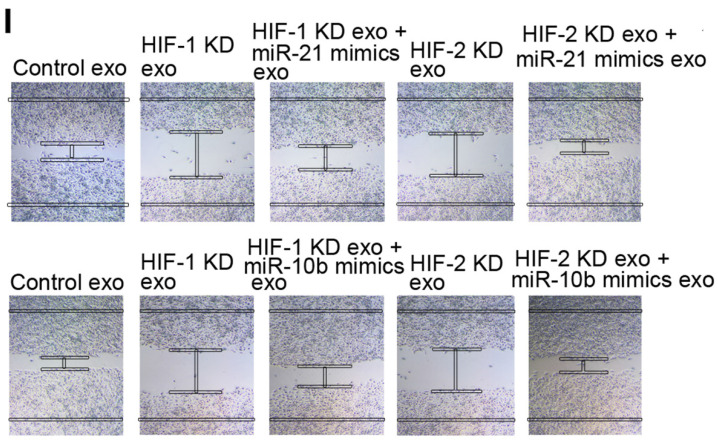
miR-21 and miR-10b mediated acidic exosome-induced cell proliferation, migration, and invasion. B, D, G and I, scratch assay; C, E, H and J, invasion assay of cancer cells modified as depicted in the Figure. Experiments performed in triplicates. *, *P* < 0.05.
